# Impact of Physical Activity on Cancer-Specific and Overall Survival of Patients with Colorectal Cancer

**DOI:** 10.1155/2013/340851

**Published:** 2013-10-03

**Authors:** Gaetan Des Guetz, Bernard Uzzan, Thierry Bouillet, Patrick Nicolas, Kader Chouahnia, Laurent Zelek, Jean-François Morere

**Affiliations:** ^1^Department of Medical Oncology, APHP, Avicenne Hospital, 125 route de Stalingrad, 125 rue de Stalingrad, 93009 Bobigny, France; ^2^Department of Pharmacology, APHP, Avicenne Hospital, 125 route de Stalingrad, 125 rue de Stalingrad, 93009 Bobigny, France; ^3^Department of Radiotherapy, APHP, Avicenne Hospital, 125 route de Stalingrad, 125 rue de Stalingrad, 93009 Bobigny, France

## Abstract

*Background*. Physical activity (PA) reduces incidence of colorectal cancer (CRC). Its influence on cancer-specific (CSS) and overall survival (OS) is controversial. *Methods*. We performed a literature-based meta-analysis (MA) of observational studies, using keywords “colorectal cancer, physical activity, and survival” in PubMed and EMBASE. No dedicated MA was found in the Cochrane Library. References were cross-checked. Pre- and postdiagnosis PA levels were assessed by MET. Usually, “high” PA was higher than 17 MET hour/week. Hazard ratios (HRs) for OS and CSS were calculated, with their 95% confidence interval. We used more conservative adjusted HRs, since variables of adjustment were similar between studies. When higher PA was associated with improved survival, HRs for detrimental events were set to <1. We used EasyMA software and fixed effect model whenever possible. *Results*. Seven studies (8056 participants) were included, representing 3762 men and 4256 women, 5210 colon and 1745 rectum cancers. Mean age was 67 years. HR CSS for postdiagnosis PA (higher PA versus lower) was 0.61 (0.44–0.86). The corresponding HR OS was 0.62 (0.54–0.71). HR CSS for prediagnosis PA was 0.75 (0.62–0.91). The corresponding HR OS was 0.74 (0.62–0.89). *Conclusion*. Higher PA predicted a better CSS. Sustained PA should be advised for CRC. OS also improved (reduced cardiovascular risk).

## 1. Introduction

Physical inactivity and obesity have been shown to increase the incidence of several cancer types, especially colorectal cancer (CRC) [1] and breast cancer [2]. CRC is among the most common cancers both in men and women, especially in developed countries [3]. There is good evidence that recreational physical activity (PA) exerts preventive effects on the development of CRC [3]. Recently, a meta-analysis (MA) assessing 52 observational studies found an inverse relationship between level of PA and incidence of CRC. The overall relative risk was 0.76 (95% confidence interval or CI: 0.72–0.81) [4]. Among patients with invasive nonmetastatic CRCs, the association between PA level on one hand and cancer specific survival (CSS) or overall survival (OS) on the other hand is less clear. Some studies assessed prediagnosis levels of PA, and others assessed postdiagnosis levels of PA, which seems to better correspond to the search for risk factors whose modification might improve CRC survival. Accordingly, expert committees have provided PA guidelines for cancer survivors, actually similar to those for primary cancer prevention (more than 150 minutes/week of moderate-to-vigorous PA) [5–8]. Considering the absence of published randomised controlled trials of the impact of PA on survival and the scarcity of prospective observational studies on this topic, we decided to perform the present MA, whose aim was to assess the influence of pre- and postdiagnosis levels of PA on CSS and OS. We chose CSS as the main outcome since OS is expected to improve among CRC survivors independently of a specific effect on cancer because PA should act favourably on associated cardiovascular risk factors in CRC patients.

## 2. Materials and Methods

### 2.1. Study Selection

We performed our MA according to a predefined written protocol. To be eligible, studies had to deal with the influence of pre- or postdiagnosis levels of PA (assessed using metabolic equivalent tasks (MET)) on CSS and OS among invasive nonmetastatic CRC patients. Across the various studies assessed, high PA levels usually corresponded to more than 17 MET hour/week.

Publications were identified by an electronic search using online PubMed, updated on February 18th, 2013, with the following keywords employed simultaneously: “colorectal cancer, physical activity, and survival.” An EMBASE query was also performed, which did not bring any additional original reference. We also searched for a systematic review on this topic in the Cochrane Database of Systematic Reviews but did not find such publication. In addition, all available references were cross-checked. Each article was carefully read by two reviewers (GDG putting the emphasis on oncological issues and BU putting the emphasis on methodological issues). For each study, both reviewers independently filled in a predefined form. Disagreements were resolved by discussion between both reviewers.

### 2.2. Statistical Analysis

All selected studies directly provided hazard ratios (HRs) with their 95% confidence interval (CI). Thus, in none study, we had to calculate HRs from the numbers of events (cancer-specific deaths, overall mortality). We chose to pool adjusted HRs, since the variables of adjustment were almost identical for all studies (age, gender, BMI, tobacco use, alcohol, and red meat consumptions) and also since, rather curiously, adjusted HR values were more conservative than raw HRs. By convention, when higher PA levels were associated with improved survival compared with lower PA levels, HRs for detrimental events were set to be inferior to 1. We used EasyMA.Net software (http://www.spc.univ-lyon1.fr/easyma.net/), available online (Department of Clinical Pharmacology, Cardiology Hospital, Lyon, France); PN performed the statistical analysis. We used a fixed-effect model (Mantel Haenszel) whenever possible and a random-effect model only in case of between-study heterogeneity. *P* values lower than 0.05 were considered as statistically significant.

## 3. Results

Our PubMed query retrieved 190 references. Of these, only 7 references corresponded to original publications fulfilling our inclusion criteria [9–15]. In addition, an eighth reference meeting our inclusion criteria was found [16], but we realised that it corresponded to a preliminary publication of the same series as in the study by Wolin et al. [16]. The main characteristics of these 7 original studies are provided in Table 1. They were published from 2006 to 2013 and included 8056 participants with CRC (3762 males and 4256 females, 5210 colon cancers and 1745 rectum cancers, with many missing data). Mean age was 67 years (range 21–82 years). One of these publications included only male patients [12], two included only female patients [10, 13], and the remainder included both male and female patients, usually without separate results according to gender. Therefore, only overall results including both men and women are provided. As expected, the cut-off value between higher and lower levels of PA varied from one study to another. However, in 3 of the 7 studies included, the cut-off value for PA level was similarly set to 18 MET hour/week. One study used a lower cutoff (8.75 MET hour/week) [9], and another study used a higher cutoff (27 MET hour/week) [12]. The two last studies did not express the PA level in terms of MET hour/week but used a simplified questionnaire [11, 15] (Table 1).

Four studies assessed the relationship between PA practised before CRC diagnosis and survival. Only one of these studies [15] found a statistically significant association between PA level and CSS. Overall, HR CSS for higher PA levels compared to lower PA levels amounted to 0.75 (0.62–0.91; fixed-effect model; *P* < 0.001), meaning that higher PA before CRC diagnosis significantly decreased by 25% the mortality related to CRC. In addition, only 2 out of the 4 individual studies assessing the relationship between PA levels and OS found a statistically significant association between higher PA levels and increased overall survival (OS). The present MA found that OS was significantly improved in case of higher PA levels (HR OS = 0.74, 0.63–0.86, fixed-effect model; *P* < 0.001).

**Figure 1:**
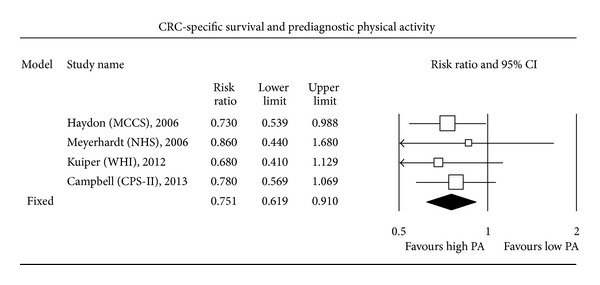


**Figure 2:**
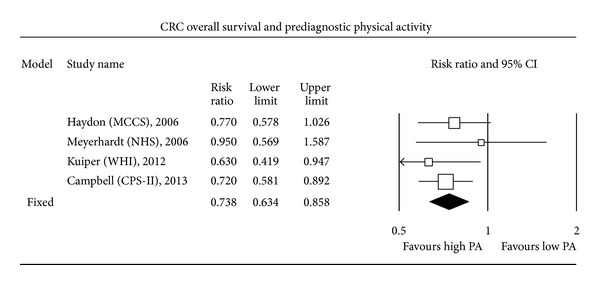


**Figure 3:**
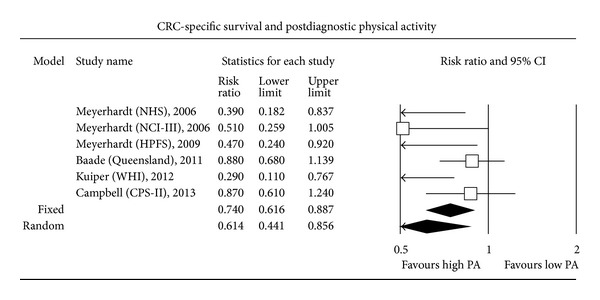


**Figure 4:**
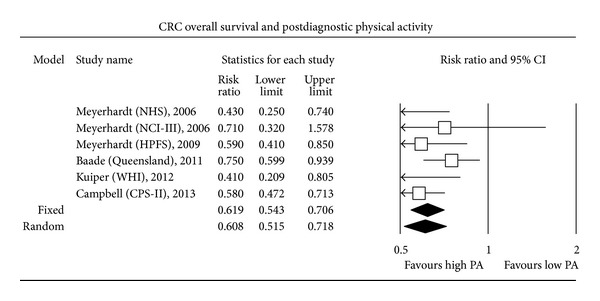


The most expected result of this MA concerned the search for a relationship between PA performed after CRC diagnosis and CSS. Only 3 out of the 6 individual studies assessing postdiagnosis PA found a statistically significant increase in CSS among patients with a high level of PA compared to patients with a low level. Overall, higher postdiagnosis PA was significantly associated with an improved CSS (HR CSS = 0.61, 0.44–0.86; random-effect model; *P* < 0.001). A random-effect model was used since a fixed-effect model led to statistically significant between-study heterogeneity. Finally, higher postdiagnosis PA level was associated with a significantly increased OS (HR OS = 0.62, 0.54–0.71; fixed-effect model; *P* < 0.001). Five out of 6 individual studies assessing the relationship between PA level and OS found a statistically significant increase in OS among patients with higher postdiagnosis PA levels (Figures 1, 2, 3, and 4).

## 4. Discussion

The present MA is, to our knowledge, the first to explore the relationships between pre- and postdiagnosis PA and CSS and OS among CRC patients. Higher postdiagnosis PA levels were associated with a better CSS, suggesting that sustained PA should be advised to nonmetastatic CRC patients. OS also significantly improved, as could be inferred since PA is expected to improve cardiovascular risk factors, independently of a specific effect on CSS. Meta-analysis is an important tool for revealing trends not always elicited in one single study. However, in the present MA, the extent of the conclusions is somewhat limited by the small number of published studies included, which increases the risk for publication bias. This MA has several limitations. It only included observational studies and was not performed on patient-level data. Details on treatments were lacking in the studies, increasing uncertainties about uncontrolled factors susceptible to influence survival. It is highly improbable that treatment differed according to prediagnosis PA level, but it is conceivable that response to treatment could differ and that heavier treatments could impair the capacity for patients to achieve higher PA levels. Conversely, physically active CRC patients would be more prone to tolerate and complete treatment, which might improve their survival. However, in almost all studies, HRs were adjusted for stage at diagnosis, a major prognostic factor. Although attempts for standardisation have been made, quantification of the level of PA practised by individual patients is not always easy since it is self-reported by CRC patients [17]. Elsewhere, an inherent bias reflecting inverse causality may exist: CRC patients with poor underlying health or a poor prognosis are expected to achieve lower PA levels. However, stage IV patients were usually excluded from the studies. It should also be stressed that the level of PA found to be associated with an increased survival was rather high (18 MET hour/week). This level would not be always easily achievable by CRC patients. 

Obviously, obesity represents a confounding factor in the relation between physical activity and CRC survival, since it probably increases the risk for CRC and it is also a limitation for practising physical activity. However, it should be mentioned that all studies included in this MA presented results adjusted for BMI. This should lessen the relevance of this confounding factor in the interpretation of the results of this MA.

Finally, considering the increased age of new incident CRC cases and the frequent comorbidities associated with this pathology, not all patients would be able to benefit from high PA.

Higher prediagnosis PA levels were associated in the present MA with a statistically significant increase in CSS and OS. These findings are in keeping with the WHO recommendations of at least 30 minutes of moderate PA 5 days per week (corresponding to 8.75 MET hour/week) in order to decrease by about 25% the risk of developing breast or colorectal cancers [5].

We chose to pool adjusted HRs instead of raw HRs since the variables of adjustment were very similar from one study to another and since, rather curiously, adjusted HRs were often more conservative than nonadjusted HRs, when compared in keeping with the tested hypotheses.

Three out of 7 studies included in this MA shared a common cutoff for PA level. This finding strengthens the validity of the conclusions. About the two studies which did not express PA levels using MET hour/week but only used a simplified questionnaire [11, 15], it is generally accepted that recollection of historical prediagnosis PA has low levels of repeatability [18]. However, allocating participants to distinct categories of PA provides a higher level of repeatability [18].

Reasons for a positive association between recreational PA and CSS are not straightforward. Perhaps PA might favour the development of less aggressive tumours, whatever the disease stage, and would decrease the formation of micrometastases. On the other hand, PA might improve the capacity of patients to tolerate surgery and adjuvant chemotherapy. Conceivably, PA might also interfere with inflammatory processes or hormonal pathways related to tumour growth (e.g., PA decreases insulin resistance). Increased plasma levels of insulin or insulin-like growth factors might predict a decrease in CSS and OS among CRC patients. Adipokines such as adiponectin and leptin might also influence CRC risk. The fact that a high PA level was significantly at least equally associated with an increased OS than with an increased CSS argues for a favourable role of PA in improving survival from diseases independent from cancer, mainly cardiovascular diseases. It should be reminded that PA is associated with a decreased mortality from several chronic diseases [19].

In conclusion, the present MA showed that higher PA levels practised both before and after CRC diagnosis predicted improved CSS and OS. Although prospective data on the benefits of PA on CRC survivors are scarce, CRC survivors should be counselled whenever possible to follow a physically active lifestyle with the reasonable goal of achieving at least 150 minutes per week of PA of moderate intensity, such as walking. These data support the need for randomised controlled trials to assess the effect of recreational PA on survival of CRC patients. A randomised intervention program evaluating this issue among high-risk stage II and stage III CRC patients is ongoing from 2008 [20].

## Figures and Tables

**Table 1:** Main characteristics of the 8 studies included in the meta-analysis.

First author Year (reference)	Number of CRCs cohort	Males/females	Colon/rectum	Variables of adjustment	Prediagnosis PA	Postdiagnosis PA
Campbell et al., 2013 [9]	2293 CPS-II	1271/991	1664/598	Age, gender, stage, BMI, and tobacco	≥8.75 MET hour/wk (*n* = 1064) versus <3.5 MET hour/wk (*n* = 255) RR CSS = 0.78 (0.57–1.08) RR OS = 0.72 (0.58–0.89)	RR CSS = 0.87 (0.61–1.24) RR OS = 0.58 (0.47–0.71)

Kuiper et al., 2012 [10]	1339 WHI	0/1339	404/96	Age, time from diagnosis, BMI, stage, alcohol, tobacco, and HRT	≥18 MET hour/wk (*n* = 277) versus no PA (*n* = 234) HR CSS = 0.68 (0.41–1.13) HR OS = 0.63 (0.42–0.96)	HR CSS = 0.29 (0.11–0.77) HR OS = 0.41 (0.21–0.81)

Baade et al., 2011 [11]	1825 Queensland	1089/736	1163/662	Age, gender, BMI, stage, tobacco, and type of therapy		593 sufficiently active versus 748 sedentary HR CSS = 0.88 (0.68–1.15) HR OS = 0.75 (0.60–0.94)

Meyerhardt et al., 2009 [12]	668 HPFS	661/0	390/110	Age, stage, colon/rectum, BMI, and tobacco		≥27 MET hour/wk (*n* = 252) versus <3 MET hour/wk (*n* = 102) HR CSS = 0.47 (0.24–0.92) HR OS = 0.59 (0.41–0.86)

Meyerhardt et al., 2006 [13]	573 NHS	0/573	421/104	Age, gender, BMI, stage, colon/rectum, CT, tobacco, and time from diagnosis	≥18 MET hour/wk (*n* = 161) versus <3 MET hour/wk (*n* = 142) HR CSS = 0.86 (0.44–1.67) HR OS = 0.95 (0.57–1.59)	≥18 MET hour/wk (*n* = 144) versus <3 MET hour/wk (*n* = 167) HR CSS = 0.39 (0.18–0.82) HR OS = 0.43 (0.25–0.74)

Meyerhardt et al., 2006 [14]	832 Stage III NCI	471/361	832/0	Age, gender, invasion, perforation, obstruction, CEA, BMI, CT arm, and PS		18–27 MET hour/wk (*n* = 84) versus <3 MET hour/wk (*n* = 273) HR RFS = 0.51 (0.26–1.01) HR OS = 0.71 (0.32–1.59)

Haydon et al., 2006 [15]	526 MCCS	270/256	336/175	Age, gender, BMI, and stage	PA (*n* = 229) versus no PA (*n* = 297) HR DFS = 0.73 (0.54–1.00) HR OS = 0.77 (0.58–1.03)	

CRC means colorectal cancer, PA: physical activity, MET: metabolic equivalent task, BMI: body mass index, CSS: cancer-specific survival, OS: overall survival, HRT: hormone replacement therapy, na: not available, CEA: carcinoembryonic antigen, CT: chemotherapy, and PS: performance status.
